# Overexpression of pressure-responsive miRNA-5703 inhibits pressure-induced growth and metastasis of liver cancer

**DOI:** 10.7150/jca.64926

**Published:** 2022-01-01

**Authors:** Si Shen, Wenli Zhou, Ji Xuan, Weijun Xu, Huabing Xu, Miaofang Yang, Liang Zhu, Zhuoxin Yang, Benzhao Yang, Bin Shi, Ying Zhao, Fangyu Wang

**Affiliations:** 1 Jinling Hosp Dept of Gastroenterology and Hepatology, Nanjing Univ, Sch Med, Nanjing 210002, P R China.; 2 Changzheng Hosp Dept of Gastroenterology, Naval Med Univ, Shanghai 200003, P R China.; 3 Changzheng Hosp Dept of Oncology, Naval Med Univ, Shanghai 200003, P R China.; 4 Dept of Cardiology, Naval Medical Center, Naval Med Univ, Shanghai 200005, P R China.; 5 Changzheng Hosp Dept of Traditional Chinese Medicine, Naval Med Univ, Shanghai 200003, P R China.

**Keywords:** liver cancer, portal hypertension, pressure, proliferation, metastasis, miRNA-5703

## Abstract

A vast majority of liver cancers coexist with cirrhosis and/or portal hypertension. A high-pressure tumour microenvironment may lead to malignant progression of liver cancer. Through quantitative reverse transcription-polymerase chain reaction, we found that miRNA-5703 was expressed at low levels in HepG2 and Huh-7 cells and pressure-treated MHCC97H implanted mouse cancer tissues, while its potential target gene, sarcoma gene (SRC), was highly expressed. The expression of miRNA-5703 was higher in liver cancer tissues from Barcelona Clinic Liver Cancer (BCLC) stage A1 patients than those from BCLC stage A2-D patients, whereas SRC showed the opposite expression pattern. Bioinformatics analysis, luciferase reporter assay, and western blotting were performed to verify the relationship between miRNA-5703 and its potential target SRC. Using intravital imaging and immunohistochemistry, we demonstrated that pressure promotes tumour growth in subcutaneous tumourigenesis nude mice, and overexpression of miRNA-5703 significantly downregulated Ki67 and upregulated NM23 in tumour tissues of mice, implying the blockage of tumour growth and metastasis. The activation of proliferation, migration, and invasion of HepG2 and Huh-7 cells by pressure, and inhibition by overexpressing miRNA-5703 were observed by cell counting kit-8 assay, flow cycle assay, transwell assay, and wound healing assay. After the intervention of pressure, inhibitor, and lentivirus to hepatoma cells, SRC, focal adhesion kinase (FAK), phosphatidylinositol 3-kinase (PI3K), serum/glucocorticoid regulated kinase-3 (SGK3), phosphoinositide dependent protein kinase 1 (PDK1), and paxillin were upregulated, and forkhead box O1 (FOXO1) and cyclin dependent kinase inhibitor 1B (P27^Kip1^) were downregulated in pressure-loaded hepatoma cells, which could be reversed by overexpression of miRNA-5703 or SRC knockdown. In conclusion, upregulation of miRNA-5703 inhibited pressure-induced growth and metastasis by suppressing the SRC-FAK-FOXO1 axis and SRC-paxillin axis. This novel perspective may be conducive to the mechano-inspired anticancer drugs of liver cancer.

## Introduction

Liver cancer is currently the third leading cause of cancer-related deaths worldwide 1. The mortality rate of liver cancer is 8.2% in both sexes combined 2. In recent years, immunosuppressants have attracted much attention in the treatment of liver cancer, but compared to other cancers, the response rate of liver cancer is much lower 3. The tumour microenvironment (TME) is a key interfering factor in immunotherapy for liver cancer 4, and the interdependence of biological, chemical, and physical cues affect the TME 5. However, the role of physical cues in liver cancer has not been definitively elucidated.

Portal hypertension (PHTN) is a recognised risk factor 6 and an independent predictor of liver cancer 7, 8. The incidence of liver cancer and mortality due to liver cancer in patients with PHTN is greater than in patients without PHTN 6. In a median follow-up of 58 months, 12.2% of patients with cirrhosis and PHTN (without varicose veins) developed liver cancer, and receiver operating characteristic curves identified that those who had a hepatic venous pressure gradient above 10 mmHg had a 6-fold increase in liver cancer incidence 8. PHTN has also been proven to be a notable risk factor for late recurrence after hepatectomy (hazard ratio: 2.424; confidence interval: 95%, 1.644-3.574; P < 0.001) 9. Recently, an increasing number of investigations using non-invasive examinations, such as computed tomography 10 and spleen stiffness measurement 7, have helped to assess the degree of PHTN and predict the occurrence, development, and recurrence of liver cancer. It has been hypothesised that modifications to the TME associated with PHTN might increase the growth potential of liver cancer and maintain a certain level of resistance to chemoembolisation or promote processes of evasion 6. PHTN increases the biological pressure in hepatic sinuses 11-13, leading to higher pressure on cancer cells 14, and destroys the mechanical balance of the TME. The mechanism by which pressure regulates the proliferation and metastasis of hepatoma cells remains poorly understood.

Pressure has been shown to be an important factor that influences the growth or aggression of solid tumours 15, 16. Zeng et al. suggested that pressure activation of malignant colon cells accelerated tumour development 17. Basson et al. applied increasing pressure between 0-80 mmHg to breast cancer cell lines (MCF-7), prostate cancer cell lines (MLL, PC3), and colon cancer cell lines (SW620, Caco-2, CT-26) for 24 h, and observed that the proliferation of all these pressurised cell lines was stimulated 18. Goetz et al. found that mechanical transduction, which is dictated by the forces generated by intercellular adhesion, cell contraction, and TME, is a key determinant of tumour progression 19. Fernnde-zsnchez et al. identified that the mechanical pressure exerted by tumour growth onto non-tumourous adjacent epithelium increased the crypt enlargement accompanying the formation of early tumourous aberrant crypt foci, which suggests that mechanical activation of signalling pathways may function in tumour heterogeneity 20. However, there have been no reports concerning the function of mechanical pressure in liver cancer.

Previous studies on mechanical-responsive miRNAs have mainly focused on cardiovascular and orthopaedic diseases. Mechanical-sensitive miR-181b-5p expression was suppressed by shear stress, which elevated levels of its target gene STAT, leading to the onset of atherosclerosis 21. miRNA-181a inhibits inflammatory responses in mice with intervertebral disc degeneration by inhibiting the ERK pathway 22. Abnormal compressive forces could regulate the expression of miRNA-221, -222, -21, and -27 in articular cartilage, and the identification of these forces has the potential to improve understanding how they impact tissue homeostasis 23. Determining whether pressure-responsive miRNAs are involved in the development of liver cancer is is an innovative project worthy of exploration.

Autophosphorylation of SRC (Y418) is an early response to mechanical transduction and leads to the activation of FAK (Y397). This finding suggests that SRC may be a messenger to initiate and spread the growth signal of cells under stress stimulation, and FAK is one of its downstream targets 24. PI3K catalyzes the phosphorylation of phosphatidylinositol-4,5-diphosphate (PIP2) to phosphatidylinositol-3,4,5-triphosphate (PIP3). PIP3 binds to the signal protein PDK1 containing a PH1 hydrophobic structure. PDK1 promotes the phosphorylation of SGK3 T-ring residues in the phosphorylation tank and activates SGK3 25. As a gene that inhibits proliferation, FoxO1 phosphorylation is an inactivation process, which promotes cell proliferation. FoxO1 has a positive regulatory effect on P27kip1. P27kip1 is a cell cycle regulator encoded by CDKN1B 26. For a long time, its function has been related to inhibiting the process of the cell cycle. Under integrin-binding or growth factor stimulation, paxillin is phosphorylated by FAK and Src to create the binding site of binding protein Crk 27. Paxillin promotes cell adhesion and movement through hyperphosphorylation of casein/serine phosphorylation sites 28. Depending on the cell environment, these activities may lead to cancer and stimulate cancer progression and metastasis.

In an earlier report 14, we concluded that exerting a pressure of 15 mmHg on liver cancer cell lines (HepG2, Huh-7, and MHCC97H) for 24 h could promote the proliferation, migration, and invasion of the cells. Under this pressurisation, five pressure-responsive miRNAs and 10,150 mRNAs were screened by miRNA and mRNA microarray, respectively. In this study, we investigated the pathophysiological role and possible mechanism of pressure-responsive miRNA-5703 in the growth and metastasis of liver cancer, using *in vivo* and *in vitro* experiments, and observed that overexpression of miRNA-5703 may suppress the mechanical-dependent development of liver cancer, which provides a theoretical basis for the combined application of miRNA-5703 overexpression with immunosuppressants.

## Materials and Methods

### miRNA and mRNA expression profiling

The differentially expressed miRNAs and mRNAs screened from the pressurised and unpressurized HepG2 cells are available in the Gene Expression Omnibus (GEO) repository (www.ncbi.nlm.nih.gov/geo/query/acc.cgi?acc=GSE119881 and www.ncbi.nlm.nih.gov/geo/query/acc.cgi?acc=GSE120194). The Kyoto Encyclopedia of Genes and Genomes (KEGG) database (www.genome.jp/kegg/annotation) was used for genome/meta genome annotation, and a bubble chart was produced using KOBAS3.0 (Peking University, Beijing, China).

### Cell culture and reagents

The HepG2 cell line was purchased from American Type Culture Collection (HB-8065; ATCC, Manassas, VA, USA). The Huh-7 cell line was purchased from the Japanese Collection of Research Bioresources Cell Bank (0403; JCRB, Manassas, VA, USA). MHCC97H and HL-7702 cell lines were purchased from the Shanghai Cell Bank of the Chinese Academy of Sciences (HB-8065; Shanghai, China). No infection was detected in any of the cells lined by mycoplasma testing. Short tandem repeat analysis was used to authenticate the cell lines. All cells were cultured at 37 °C under 5% CO_2_ in Dulbecco's Modified Eagle's Medium (DMEM; HyClone, Logan, UT, USA) containing 10% foetal bovine serum (Gibco Life Technologies, Carlsbad, CA, USA), 1 mM sodium pyruvate, 2 mM glutamine, 100 U/mL penicillin G, 100 mg/mL streptomycin, and 10 mM HEPES (Sigma, San Francisco, CA, US). Cells were treated with 0.2% trypsin and 0.02% EDTA (Thermo Fisher Scientific, Waltham, MA, USA) when collected for experiments.

### Pressure loading

The 2-dimensional (2D) pressure loading system was designed and manufactured by our research group, and the protocol can be found in our previous papers 14, 29, 30. The Flexcell-5000 Compression System (Flexcell International Corp, Burlington, NC, US) was used to exert pressure on the 3D cultured cells. The methods of cell 3D culture and pressure loading have been described in our previous paper 14.

### Cell Counting Kit-8 assay

A Cell Counting Kit-8 (CCK-8) assay was conducted using a kit (Dojindo Laboratories, Kumamoto, Japan) to detect cell proliferation, and the protocol used has been detailed in our previous paper 14.

### Cell cycle analysis by flow cytometry

A cell cycle assay kit (MULTI SCIENCES, Hangzhou, Zhejiang, China) was used to detect the cell cycle, and the details of the methods have been reported in our previous paper 14.

### Transwell assay

Transwell® Permeable Supports consisting of Snapwell™ and Netwell™ inserts (Corning, Corelle, NY, US) were used to perform the cell invasion assay, and the protocol used was the same as that used in our previous paper 14.

### Wound healing assay

Here, 5 × 10^5^ pre-treated cells were seeded into each well of a 24-well plate (Corning, Corelle, New York, USA), and the details of the methods have been reported in our previous paper 14.

### Immunofluorescence staining

A cover slide was placed in each well of a 6-well plate (Corning, Corelle, New York, USA) and 7 × 10^4^ cells were added per well for overnight cultivation. Subsequently, after fixing with 4% paraformaldehyde (Sinopharm Chemical Reagent Co. Ltd., Shanghai, China) for 30 min, the cells were blocked with 3% bovine serum albumin (BSA) for 30 min after membrane permeabilisation. They were then incubated with a 1:300 dilution of primary antibodies overnight at 4 °C. Then, a 1:300 dilution of secondary antibodies were added and incubated for 1 h. DAPI (G1012, Servicebio, Wuhan, China) was used to stain nuclei. The stained cells were observed and photographed using a fluorescence microscope (DMi8, Leica, Wetzlar, Germany) after being sealed with a sealing reagent that inhibited fluorescence quenching.

### Cytoskeleton staining

For cytoskeleton staining, a cover slide was placed in each well of a 6-well plate and 7 × 10^4^ cells were added per well for overnight cultivation. The next day, the culture medium was discarded and the cells on the climbing slides were washed thrice with phosphate-buffered saline (PBS; HyClone, Logan, UT, US) for 5 min each. Then, 1 mL 4% paraformaldehyde (Sinopharm Chemical Reagent Co. Ltd., Shanghai, China) was added to the pores to fix the cells for 30 min. The cells were then washed thrice with PBS for 5 min each. The cells were stained with 80 mL 1:300 phalloidin (Servicebio, Wuhan, China) at room temperature (25 ℃) for 2 h. The cells were then washed with PBS thrice for 5 min each. Finally, 500 μL DAPI was used to stain the nuclei for 15 min. The staining solution was discarded and the cells were washed thrice with PBS for 5 min each. The slides were observed and photographed using a fluorescence microscope (DMi8, Leica, Wetzlar, Germany) after being sealed with a sealing reagent to inhibit fluorescence quenching.

### Dual-Luciferase reporter gene assay

HEK293T cells were co-transfected with pMIR-Report-SRC 3ʹ-UTR (wild-type or mutant), miR-5703 mimic, or the control. The cells were harvested 24 h later, and a luciferase assay was performed using the Dual-Luciferase Reporter Assay System (E1910, Promega, WI, US) according to the manufacturer's instructions. Luciferase activity was expressed as a percentage of that of the control. The universal sequencing forward and reverse primers of H11164 and H11165 vectors (OBIO, Shanghai, China) were Luc-C-F (5ʹ-GAGGAGTTGTGTTTGTGGAC-3ʹ) and M13F (5ʹ- TGTAAAACGACGGCCAGT-3ʹ). The binding site was a 7mer-m8 which was predicted from TargetScan website (http://www.targetscan.org/vert_71/).

### Western blotting

Cells were lysed in RIPA lysis buffer (Servicebio, Wuhan, China). The lysates were centrifuged at 16099.2 x *g* for 10 min, and the protein concentrations were measured by the standard BCA assay (Pierce^TM^ BCA Protein Assay Kit, New York, USA) according to the manufacturer's instructions. Equal amounts of protein were separated using a PAGE Gel Fast Preparation Kit (Beyotime Biotechnology, Shanghai, China) according to the manufacturer's instructions. The membranes were then blocked in PBST containing 3% BSA for 1 h at room temperature. Then, they were probed with primary antibody at 4 °C overnight and incubated with the secondary antibody for 1 h at room temperature. The protein bands were detected using the ECL western blotting Detection Reagent (Pierce, New York, USA). The film was exposed at various times depending on the antibody.

### RNA extraction and quantitative reverse transcription PCR

mRNA levels and miRNA levels were quantified by reverse transcription PCR** (**RT-qPCR**)** as previously described 14. The primers (wcgene Biotech, Shanghai, China) used in this study are listed in Table 1 and 2. Glyceraldehyde 3-phosphate dehydrogenase (GAPDH) and U6 were used as controls for mRNA and miRNA detection, respectively. Relative gene expression was calculated using the 2^-ΔΔCq^ method 31 after normalisation to the expression of GAPDH or U6 small nuclear RNA, and the results were statistically analysed as mean ± standard deviation (SD).

### Cell adhesion assay

Matrigel (Corning, Corelle, New York, USA) and serum-free DMEM medium (HyClone, Logan, UT, USA) were mixed to form a basement membrane of 10 μg/mL. Six 96-well plates (Corning, Corelle, New York, USA) were laid with 60 μL/well of the basement membrane, and the plates were placed in an ultra-clean cabinet for 24 h. Then, serum-free DMEM was added to the wells. After 1 h, the matrix adhesive Matrigel was washed away. HepG2, Huh-7, and MHCC97H cells were inoculated into six 96-well plates at a density of 4,000 cells/well. For each cell line, one plate was cultured under environmental pressure, and the other plate was pressurised to 15 mmHg for culture. After 24 h, the medium was discarded, and the cells were washed thrice with PBS (HyClone, Logan, UT, US). The cells were fixed with 100 μL methanol (Aladdin, Shanghai, China) for 15 min and then the methanol was discarded. Then, 100 μL of hoe33258 dye solution (Beyotime Biotechnology, Shanghai, China) was added, incubated for 15 min, and washed out with H_2_O_2_ (Aladdin, Shanghai, China). The number of cells was recorded and photographed using a fluorescence microscope (CX31; Olympus Corporation, Tokyo, Japan).

### Plasmid construction and lentivirus packaging

The overexpressed miRNA-5703 plasmid and sarcoma gene (SRC) intervention plasmid used for lentivirus packaging were constructed by Shanghai Collariaceae Biology (Shanghai, China). Based on the principle of base complementary pairing, three interfering target sequences were designed (5ʹ-GACAGACCTGTCCTTCAAGAA-3ʹ; 5ʹ-GCTCGGCTCATTGAAGACAAT-3ʹ; 5ʹ-GGCTCCAGATTGTCAACAACA-3ʹ) and primers were synthesised. The primers were annealed and ligated into a pLenR-GPH interference vector (Zorin, Shanghai, China). The plasmid was digested with restriction enzymes to ensure the correct size of the cleaved band. Dh5a competent cells were used to transform the interference plasmid and amplify it in bacteria. The plasmid was then extracted and sequenced.

### Tumour formation in nude mice

Twenty-four nude mice were divided into four groups, each group treated with subcutaneous injections under the armpit as follows: 1) 200 μL MHCC97H cells with a density of 1 × 10^7^ cells/mL; 2) 200 μL of MHCC97H cells at a density of 1 ×10^7^ cells/mL under 15 mmHg of pressure for 24 h; 3) 200 μL of MHCC97H cells (with empty virus vector) at a density of 1 ×10^7^ cells/mL under 15 mmHg of pressure for 24 h; and 4) 200 μL of MHCC97H cells with miRNA-5703 overexpression infected by lentivirus at a density of 1 ×10^7^ cells/mL under 15 mmHg of pressure for 24 h. All nude mice were weighed before MHCC97H cell inoculation. The vital signs of nude mice were monitored during the experiment, and *in vivo* imaging was performed on day 10 after cell inoculation.

### Immunohistochemistry

Tissue sections were rehydrated, immersed in 0.3% dH_2_O, and then treated with citrate buffer (10 mM, pH 6.0). Next, the sections were treated with primary antibodies as follows: Ki67 (1:200 dilution, Abcam, Shanghai, China) and NM23 (1:500 dilution, Abcam, Shanghai, China) overnight at 4 °C, followed by incubation with secondary antibodies for 20 min at room temperature. Sections were visualised with diaminobenzidine, diaminobenzidine substrate, and haematoxylin counterstain. Images were acquired with a Leica microscope (DMi8, Leica, Wetzlar, Germany) at × 400 magnification.

### Hematoxylin-eosin staining

Liver tissues were fixed in 4% paraformaldehyde solution for 24 h. The dehydration process was conducted with the conventional gradient alcohol for 1 min each time, followed by two 5 min washes with xylene, wax dipping, paraffin embedding, and slicing (4 μm slices). Paraffin slices were routinely dewaxed in water and cleaned with xylene for 5 min thrice, with 100% ethanol for 10 min twice, and twice with 95% ethanol for 10 min. Next, they were washed twice in dH_2_O for 5 min. The sections were stained with haematoxylin for 10 min. The slices were cleaned with dH_2_O and then differentiated with 1% hydrochloric acid alcohol for 5 s. After cleaning with dH_2_O, the slices were treated with ammonia water and stained with eosin solution for 3 min. The tissues were dehydrated with 95% ethanol twice for 5 min, and then they were placed in xylene twice for 5 min. The slides were then removed and dried. Finally, they were sealed with neutral gum, and the histopathological changes of the liver tissues were observed under an optical microscope (CX31, Olympus Corporation, Tokyo, Japan).

### Statistical analyses

Results were compared using Student's t-test, and the data are expressed as means ± SD of at least three independent experiments. All P values were calculated using two-tailed tests, obtained using the SPSS software (version 16.0; SPSS, Chicago, IL). Statistical significance was set at P < 0.05.

## Results

### Prediction of pressure-induced signalling pathways

**Figure 1:**
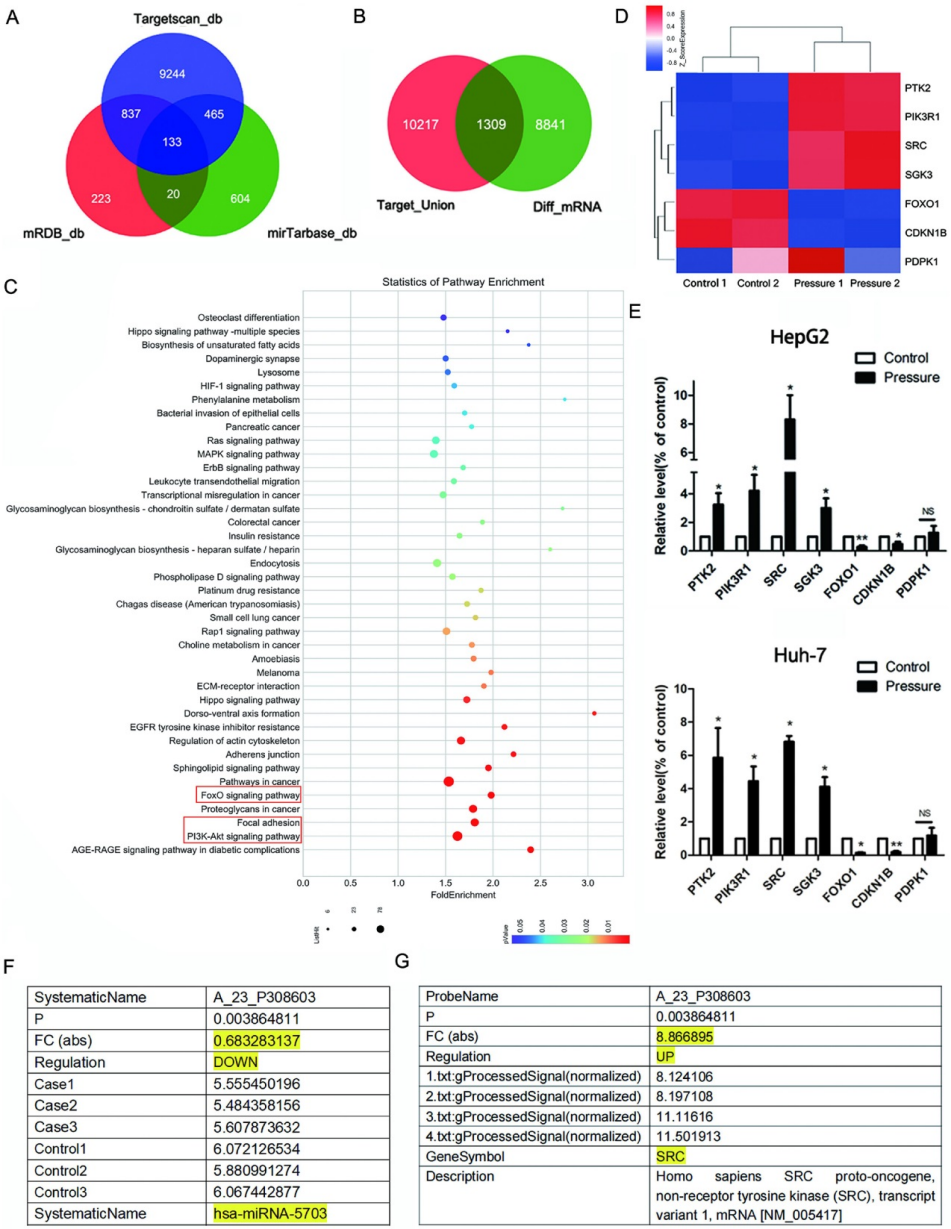
Prediction of pressure-induced signaling pathways. **(A)** The predicted overlapping target genes of differentially expressed microRNAs, identified by microarray, using three publicly available bioinformatics algorithms (TargetScan, miRDB, and miRTarBase).** (B)** The predicted target genes of the differentially expressed microRNA identified by microRNA microarray were combined and ones that corresponded with differentially expressed mRNAs were identified by mRNA microarray, and 1,309 predicted target genes were obtained. **(C)** KEGG pathway analysis was conducted on the 1,309 mRNAs, and bubble diagrams were obtained to predict pressure-induced signaling pathways. **(D)** The expression of genes associated with cancer-associated pressure-inducible pathways were verified by mRNA microarray. **(E)** The expressions of genes associated with cancer-associated pressure-inducible pathways were verified by RT-qPCR. **(F)** The table shows the result of the miRNA microarray with miRNA-5703. **(G)** The table shows the result of the mRNA microarray with SRC.* NS* indicates the means are not significantly different (P > 0.05), mean ± SD, n = 3. A two-tailed Student's t-test was used. **P < 0.01, *P < 0.05.

Using three target gene prediction websites (MicroDB, MicroTarBase and TargetScan), the target genes of five pressure-sensitive miRNAs (miRNA-5703, miRNA-630, miRNA-7641, miRNA-7-5p, and miRNA-4485-3p) 14 identified by miRNA microarray were predicted. MicroDB predicted 1,213 target genes, TargetScan predicted 10,679 target genes, and MicroTarBase predicted 1,222 target genes (Fig. **1A**). Combined, 11,526 genes were obtained. Of the 11,526 predicted target genes, 1,309 corresponded to differentially expressed mRNAs, which were identified by an mRNA microarray (Fig. **1B**). The SRC gene was found amongst the differentially expressed predicted target genes.

KEGG pathway analysis of the 1,309 target genes revealed the relative materiality of the pathways of which the top three cancer growth and metastasis-related pathways were the focal adhesion (FA) pathway, the PI3K pathway, and the FOXO pathway (Fig. **1C**). By investigating the KEGG pathway maps, we found that the conjoint upstream gene of the three pathways was SRC, which is also one of the predicted target genes of downregulated miRNA-5703 under pressure loading.

An mRNA microarray was used to detect changes in HepG2 cells cultured for 24 h (control group) and cells under 15 mmHg of pressure for 24 h (pressure-treated group). A cluster heat map of differentially expressed genes screened by microarray which correlated to the above three pathways is shown in Fig. **1D**. Among them, protein tyrosine kinase-2 (PTK2), phosphatidylinositol 3-kinase (PI3K, PIK3R1), SRC, and serum/glucocorticoid regulated kinase-3 (SGK3) were upregulated in pressurised liver cancer cells; forkhead box O1 (FOXO1) and cyclin dependent kinase inhibitor 1 B (CDKN1B, P27^Kip1^) were downregulated; phosphoinositide dependent protein kinase 1 (PDK1) showed no statistically significant changes in expression. We verified the expression of these genes in HepG2 and Huh-7 cell lines by RT-qPCR (Fig. **1E**, P < 0.05, or P < 0.01).

### Verification of SRC as a target of miRNA-5703

**Figure 2:**
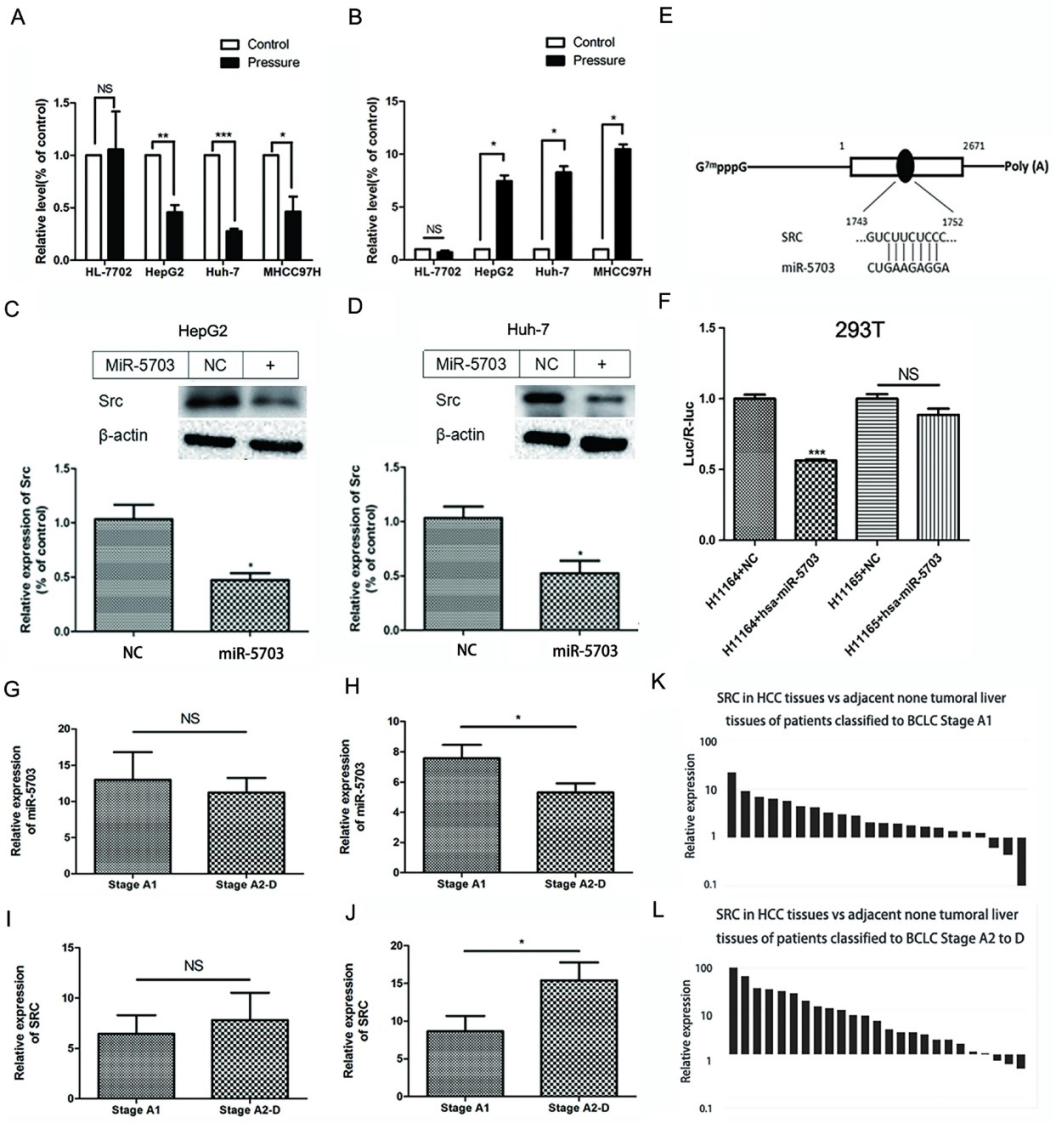
Verification of SRC as a target of miRNA-5703. **(A)** RT-qPCR analysis demonstrated that pressure inhibited the expression of miRNA-5703 in different malignant hepatoma cell lines, but had no effect on the normal liver cell line. **(B)** RT-qPCR analysis demonstrated that pressure promoted the expression of SRC in different malignant hepatoma cell lines, but had no effect on the normal liver cell line. **(C)** In HepG2 and** (D)** Huh-7 cells SRC mRNA levels were negatively regulated by overexpression of miRNA-5703. β-actin is shown as a loading control. **(E)** The binding site of miRNA-5703 in the 3ʹ-UTR of the SRC gene was predicted by TargetScan. **(F)** Luciferase reporter assays were performed in HEK293T cells cotransfected with SRC with the wild-type 3ʹ-UTR(h1164) or mutated 3ʹ-UTR(h1165) and miRNA-5703 mimics or microRNA negative control (NC). **(G)** The expression of miR-5703 in liver cancer tissues from patients in stage A2-D was suppressed relative to the expression in tissues from patients in stage A1, **(H)** while a difiference in expression was not observed between the adjacent normal tissues of patients in the two stages.** (I)** The mRNA levels of SRC in liver cancer tissues from patients in stage A2-D was higher than that in tissues from patients in stage A1 **(J)** while a difiference in expression was not observed between in the adjacent normal tissues of patients in the two stages. **(K)** and** (L)** The higher expression of SRC in HCC tissues was observed by RT-qPCR analysis. *NS* indicates the means are not significantly different (P > 0.05), mean ± SD, n = 3. A two-tailed Student's t-test was used. ***P < 0.001, **P < 0.01, *P < 0.05.

The expression of miRNA-5703 and SRC detected by miRNA array and mRNA microarray are shown in Fig. **1F** and **1G**, respectively. The expression of miRNA-5703 in pressurised HepG2 cells was downregulated by approximately 0.68 times to that in the cells of control group (Fig. **1F**), and the expression of SRC was upregulated by approximately 8.87 times to that in the cells of control group (Fig. **1G**). We then verified the microarray results in three liver cancer cell lines (HepG2, Huh-7, and MHCC97H) by RT-qPCR (Fig. **2A**, P < 0.05, P < 0.01, or P < 0.001 and **2B**, P < 0.05), and the results were consistent with those of the microarray. In addition, there was no significant change in their expression in the normal liver cell line HL-7702 (Fig. **2A**, P > 0.05, and **2B**, P > 0.05).

Western blotting showed that SRC expression was inhibited in both HepG2 and Huh-7 cells after miRNA-5703 overexpression (Fig. **2C** and** 2D**, P < 0.05). The miRNA-5703 binding site was predicted using the TargetScan website (Fig. **2E**), and it was a 7mer-m8 positioned at nucleotides 1,745-1,751 of the SRC 3¢-UTR. Wild-type plasmids and plasmids with a mutated binding site were transfected into HEK293T cells. The light signals produced by the luciferase of firefly and produced by the luciferase of sea kidney were detected, and their ratios were calculated. The ratio decreased by 43.69% after overexpression of miRNA-5703 with the wild-type binding site in SRC (Fig. **2F**, P < 0.001), while there was no significant difference in the ratio before and after overexpression of miRNA-5703 after binding site mutation (Fig. **2F**, P > 0.05).

Twenty-two cases of liver cancer tissues and adjacent normal tissues (more than 5 cm away from cancer tissues) were collected from patients with liver cancer that were classified as BCLC stage A1 (without PHTN). Twenty-five cases of liver cancer tissues and adjacent normal tissues were collected from patients with liver cancer that were classified as stage A2-D (with PHTN). The expression of miR-5703 in liver cancer tissues from patients in stage A2-D was suppressed relative to that of tissues from patients in stage A1 (Fig. **2G**, P < 0.05), but not in the adjacent normal tissues (Fig. **2H**, P > 0.05). The mRNA levels of SRC in liver cancer tissues from patients in stage A2-D was higher than that of tissues from patients in stage A1 (Fig. **2I**, P < 0.05), but not in the adjacent normal tissues (Fig. **2J,** P > 0.05). In the 22 paired tissues of patients in stage A1, 19 (86.4%) showed higher SRC expression in liver cancer tissues than in adjacent normal tissues, of which 12 (63.1%) highly expressed SRC (> 2 times) and 7 (36.8%) did not have as high expression levels (< 2 times; Fig.** 2K**). In 25 paired tissues of patients in stage A2-D, the expression of SRC in 22 (88%) of liver cancer tissues was higher than that in adjacent normal tissues, of which 19 pairs (86.3%) highly expressed SRC (> 2 times), while 3 pairs (15.7%) did not have as high expression levels (< 2 times; Fig.** 2L**).

### Overexpression of miRNA-5703 inhibits pressure-induced proliferation of hepatoma cells

**Figure 3:**
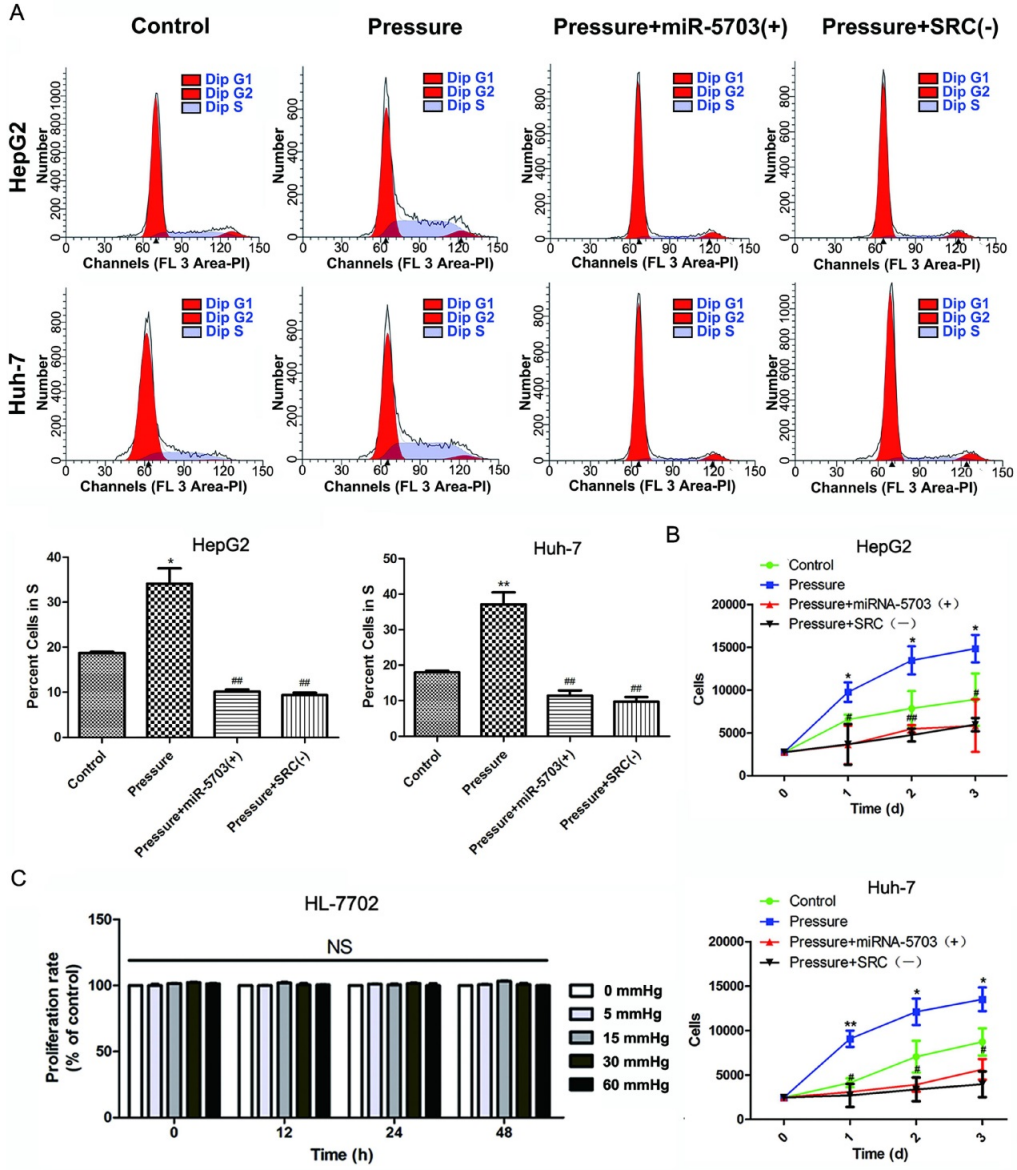
Overexpression of miRNA-5703 inhibited pressure-induced proliferation of hepatoma cells.** (A)** Overexpression of miRNA-5703 and SRC silencing reduced the number of cells in S phase, which was assessed by flow cytometry analysis. **(B)** Overexpression of miRNA-5703 and SRC gene silencing inhibited pressure-induced hepatoma cell proliferation, which was assessed by CCK-8 assay. **(C)** CCK-8 assay was used to detect the proliferation of normal hepatocyte HL-7702 under different pressure loading conditions, and no significant difference was found. *NS* indicates the means are not significantly different (P > 0.05), mean ± SD, n = 3. A two-tailed Student's t-test was used vs Control: ***P < 0.001, **P < 0.01, *P < 0.05; Vs Pressure: ^##^P < 0.01, ^#^P < 0.05.

Flow cytometry was used to detect proliferation of the HepG2 and Huh-7 cell lines. As shown in Fig. **3A**, overexpression of miRNA-5703 significantly inhibited the proportion of HepG2 and Huh-7 cells in the S phase (P < 0.01), and knocking down SRC had similar effects (P < 0.01). The HepG2 and Huh-7 cell lines were categorized into four groups based on the experimental conditions: control (conventional culture), pressure (culture under 15 mmHg of pressure), pressure + miRNA-5703 (+) (culture under 15 mmHg of pressure with over-expression of miRNA-5703), and pressure + SRC (-) (culture under 15 mmHg of pressure after knocking down SRC). The CCK-8 assay was used to detect the proliferation of the liver cancer cells (Fig.** 3B**, P < 0.05, P < 0.01). The results showed that overexpression of miRNA-5703 significantly inhibited the proliferation of HepG2 and Huh-7 cells, and knocking down SRC achieved similar results. The CCK-8 assay showed that there was no effect on the growth of pressure-loaded HL-7702 cells (Fig. **3C**, P > 0.05).

### Overexpression of miRNA-5703 inhibits pressure-induced cell proliferation via SRC-FAK--FOXO1 pathway

**Figure 4:**
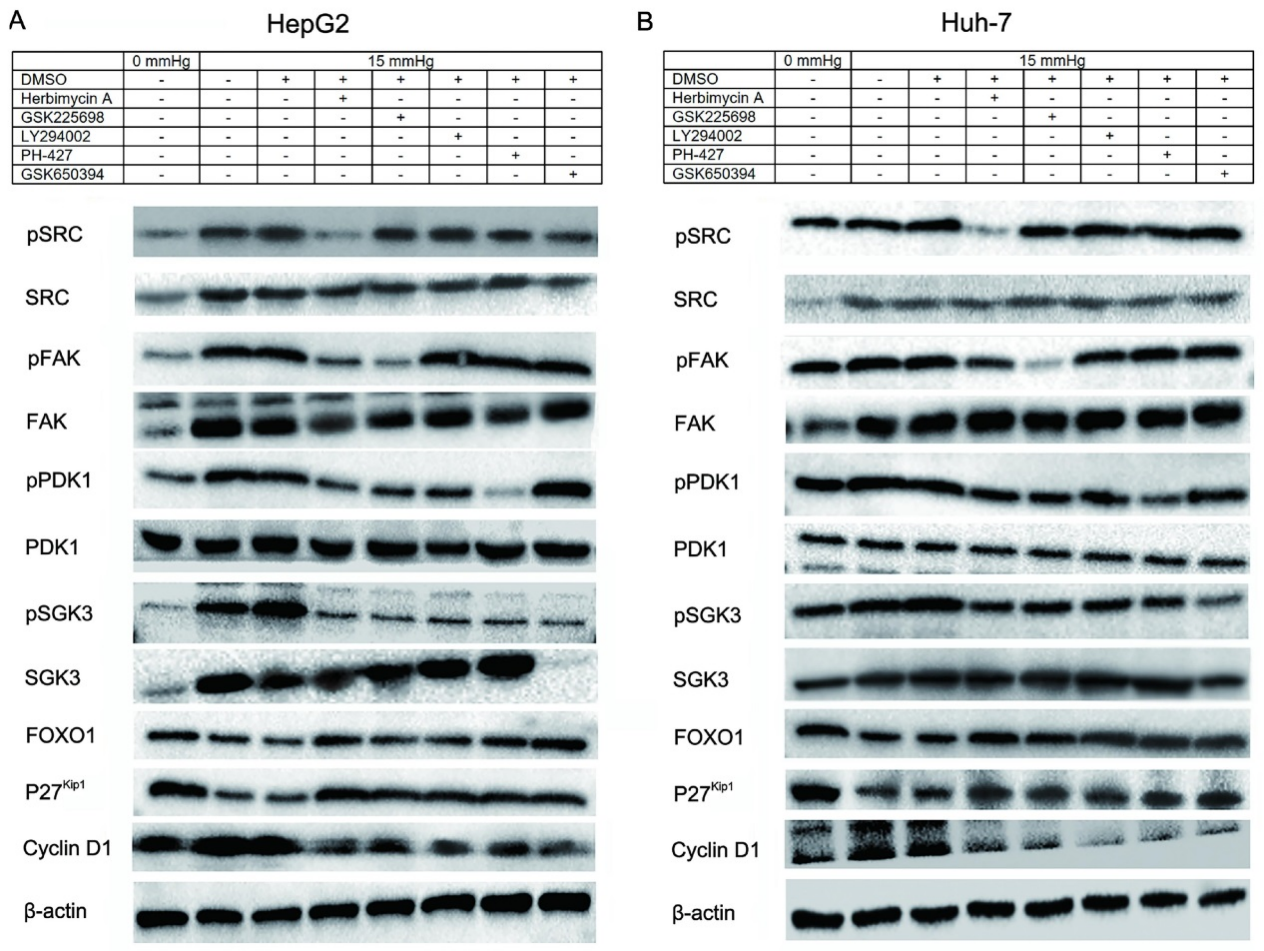
Pressure induced cell proliferation via the SRC-FAK-FOXO1 pathway. Upregulations of SRC, FAK, PI3K, SGK3, and PDK1, and downregulation of FOXO1 and p27^Kip1^ in pressure loaded cells were observed by western blotting. The upstream and downstream interaction relationship and sequence of proteins in the cascade reaction were explored through the application of inhibitors. The relationship between the inhibitors and their target proteins are as follows: Herbimycin A-SRC (Y418); LY294002-PI3K; GSK2256098-FAK (Y397); PHT-427-PDK1 (S241); EMD638683-SGK3 (T320).

Using western blotting, we found that 15 mmHg pressure loading promoted the expression of SRC, FAK, PI3K, and SGK3, and inhibited the expression of FOXO1 and P27^Kip1^ (Fig. **4A** and** 4B**), which was consistent with the results of the microarray illustrated in Fig. **1D**. Meanwhile, the expression of phosphorylated SRC (pSRC; Y418), pFAK (Y397), pPDK1 (S241), and pSGK3 (T320) were upregulated, which indicated that pressure not only increased the transcription of SRC, FAK, PIK3R1, and SGK3 at the mRNA level, but also activated the phosphorylation of these proteins. It should be noted that the expression of PDK1 at the mRNA level is not regulated by pressure, but pressure can promote phosphorylation of PDK1 at the protein level. Pressure loaded cells were treated with Herbimycin A (pSRC inhibitor), Gsk2256098 (pFAK inhibitor), LY294002 (PI3K inhibitor), PHT-427 (PDK1 inhibitor) and EMD638683 (SGK3 inhibitor; Fig. **4A** and** 4B**), and the relationship between upstream and downstream proteins in the cascade reaction was detected and the results of the grey analysis are shown as histogram in Fig. **S1** (HepG2) and Fig. **S2** (Huh-7).

**Figure 5:**
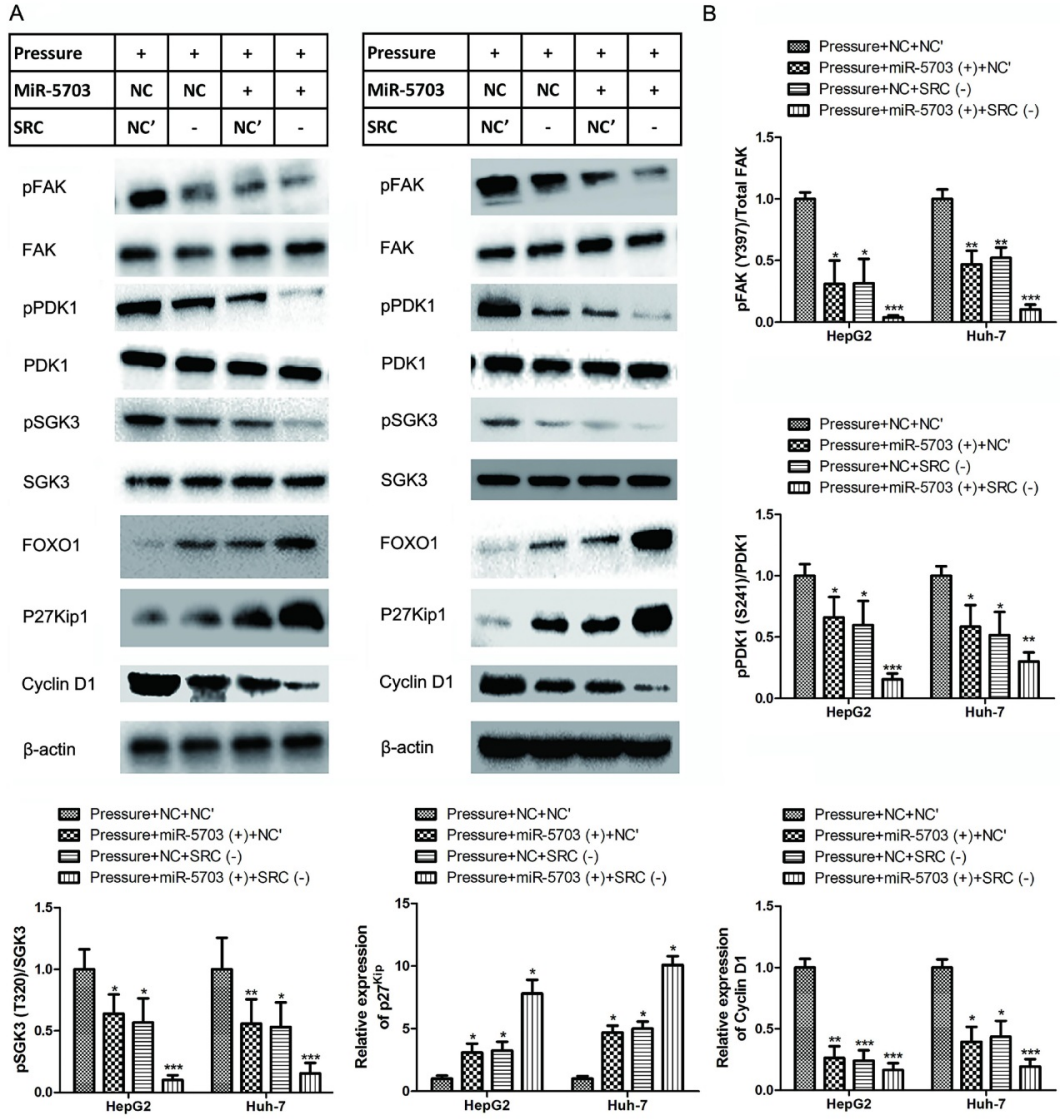
Overexpression of miRNA-5703 and SRC gene silencing reversed the proliferative effects induced by pressure loading in the indicated cells. **(A)** The expression of proteins in the SRC-FAK-FOXO1 pathway were detected by western blotting in HepG2 (left) and Huh-7 (right). β-actin was used as a loading control. **(B)** Using grey analysis, the ratio of phosphorylated protein to total protein was calculated and is displayed in a histogram, and the expression differences between the groups are shown. Mean ± SD, n = 3. A two-tailed Student's t-test was used. ^***^P < 0.001, ^**^P < 0.01, ^*^P < 0.05.

Overexpression of miRNA-5703 or SRC knockdown inhibited the expression of downstream proteins, which suggests a mechanism by which miRNA-5703 inhibits tumour growth. (Fig. **5**, P < 0.05, P < 0.01 or P < 0.01).

### Overexpression of miRNA-5703 inhibits pressure-induced migration and invasion of hepatoma cells

**Figure 6:**
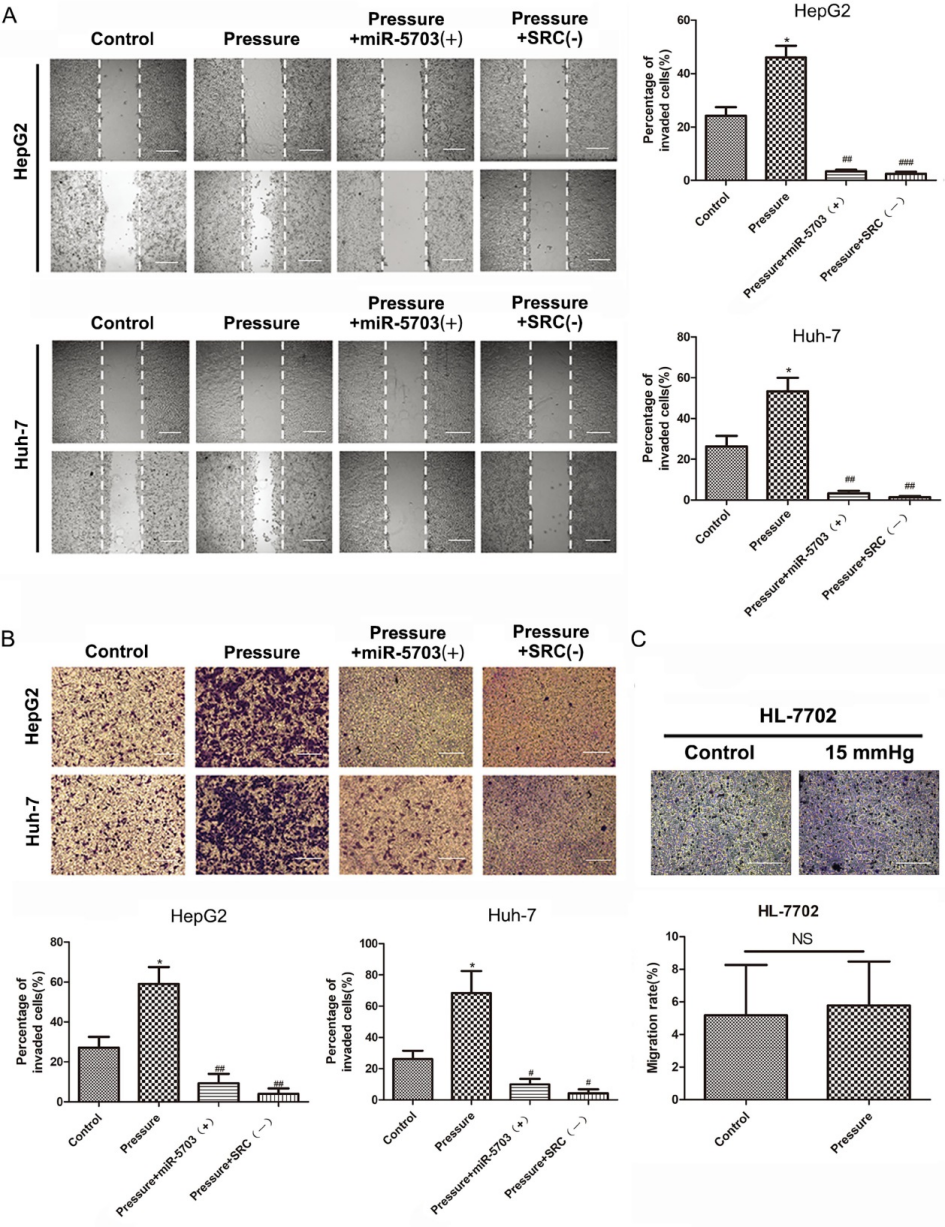
Overexpression of miRNA-5703 inhibited pressure-induced migration and invasion of hepatoma cells. **(A)** Overexpression of miRNA-5703 and SRC gene silenncing inhibited pressure-induced hepatoma cell migration which was assessed by cell scratch assay. The scale bars are 25 μm. **(B)** Overexpression of miRNA-5703 and SRC gene silencing inhibited pressure-induced hepatoma cell invasion which was assessed by transwell assay. The scale bars are 25 μm. **(C)** The transwell assay showed that there was no significant change in mobility of HL-7702 cells after pressure loading. Representative photographs were taken at a ×400 magnification. The scale bars are 25 μm. *NS* indicates the means are not significantly different (P > 0.05), mean ± SD, n = 3. A two-tailed Student's t-test was used. Vs Control: ***P < 0.001, **P < 0.01, *P < 0.05; vs Pressure: ^###^P < 0.01, ^##^P < 0.01, ^#^P < 0.05.

HepG2 and Huh-7 cell lines were divided into four groups based on experimental conditions: control (conventional culture), pressure (15 mmHg, 24 h), pressure + miRNA-5703 (+) (15 mmHg, 24 h + miRNA-5703 over-expression), and pressure + SRC (-) (15 mmHg, 24 h + SRC knockdown). Using a cell scratch assay, we showed that both overexpression of miRNA-5703 and SRC knockdown inhibited the migration of HepG2 and Huh-7 cells. (Fig. **6A**, P < 0.05, P < 0.01 or P < 0.001). A transwell assay showed that overexpression of miRNA-5703 significantly inhibited the invasion of HepG2 and Huh-7 cells, and SRC knockdown had a similar effect on the proliferation of the hepatoma cells (Fig. **6B**, P < 0.05, or P < 0.01). However, pressure had no significant effect on the migration of HL-7702, as assessed by the transwell assay (Fig.** 6C**, P > 0.05).

**Figure 7:**
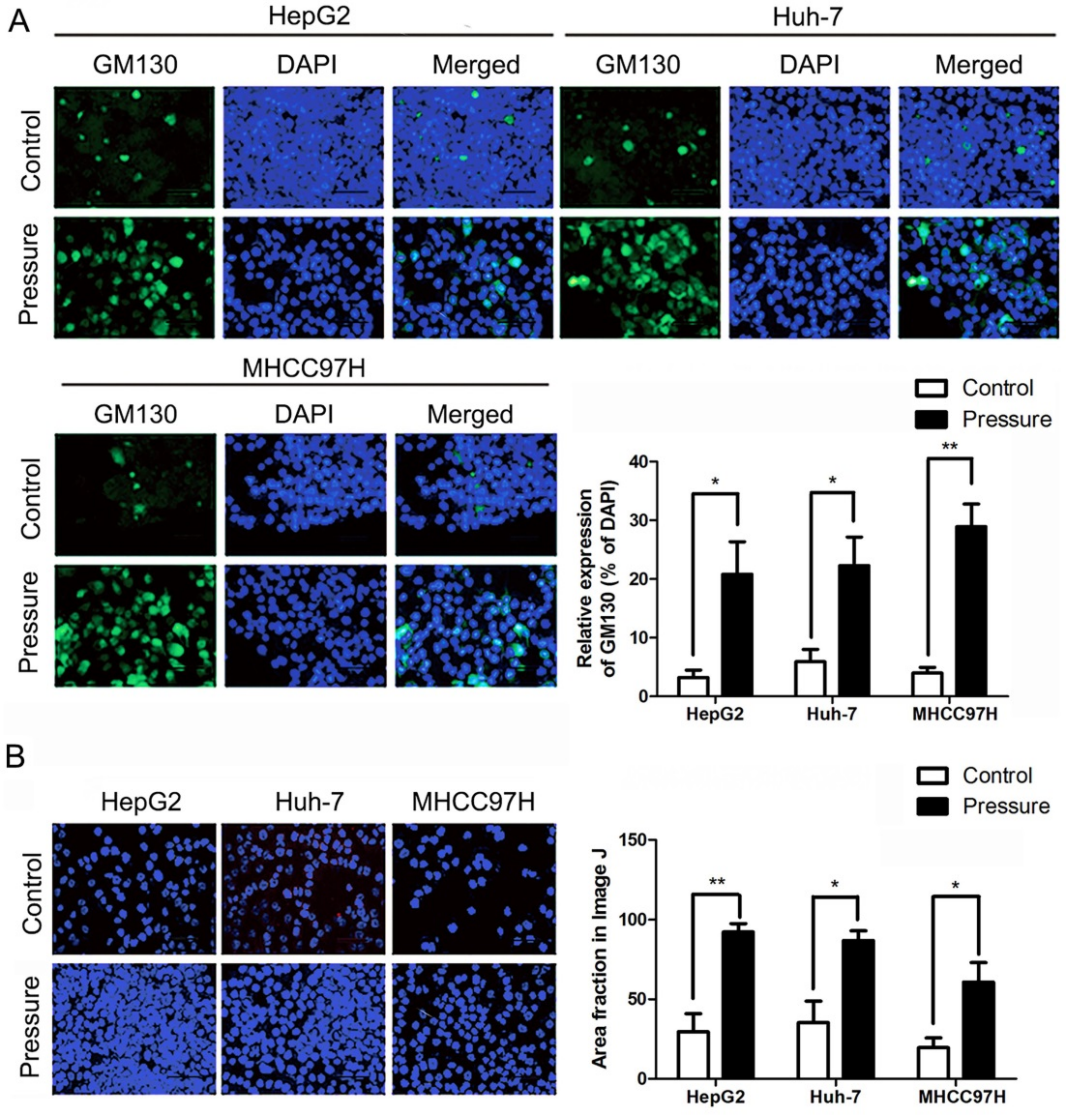
** (A)** The number of adherent HepG2, Huh-7, and MHCC97H cells on the matrix gel increased in cell adhesion assays. The scale bars are 50 μm. **(B)** GM130 expression in the indicated cells was analyzed by immunofluoresce. The scale bars are 50 μm. Mean ± SD, n = 3. A two-tailed Student's t-test was used vs Control: **P < 0.01, *P < 0.05

The peripheral membrane protein GM130, which is strongly attached to the Golgi membrane, is involved in controlling cell polarisation and directed cell migration 32. The expression of GM130 was also increased in the HepG2, Huh-7, and HL-7702 cells, which was detected by an immunofluorescence assay (Fig. **7A**). The cell adhesion assay showed that the number of HepG2, Huh-7, and MHCC97H cell lines adhering to the matrix was significantly increased after pressure loading (Fig. **7B**).

**Figure 8:**
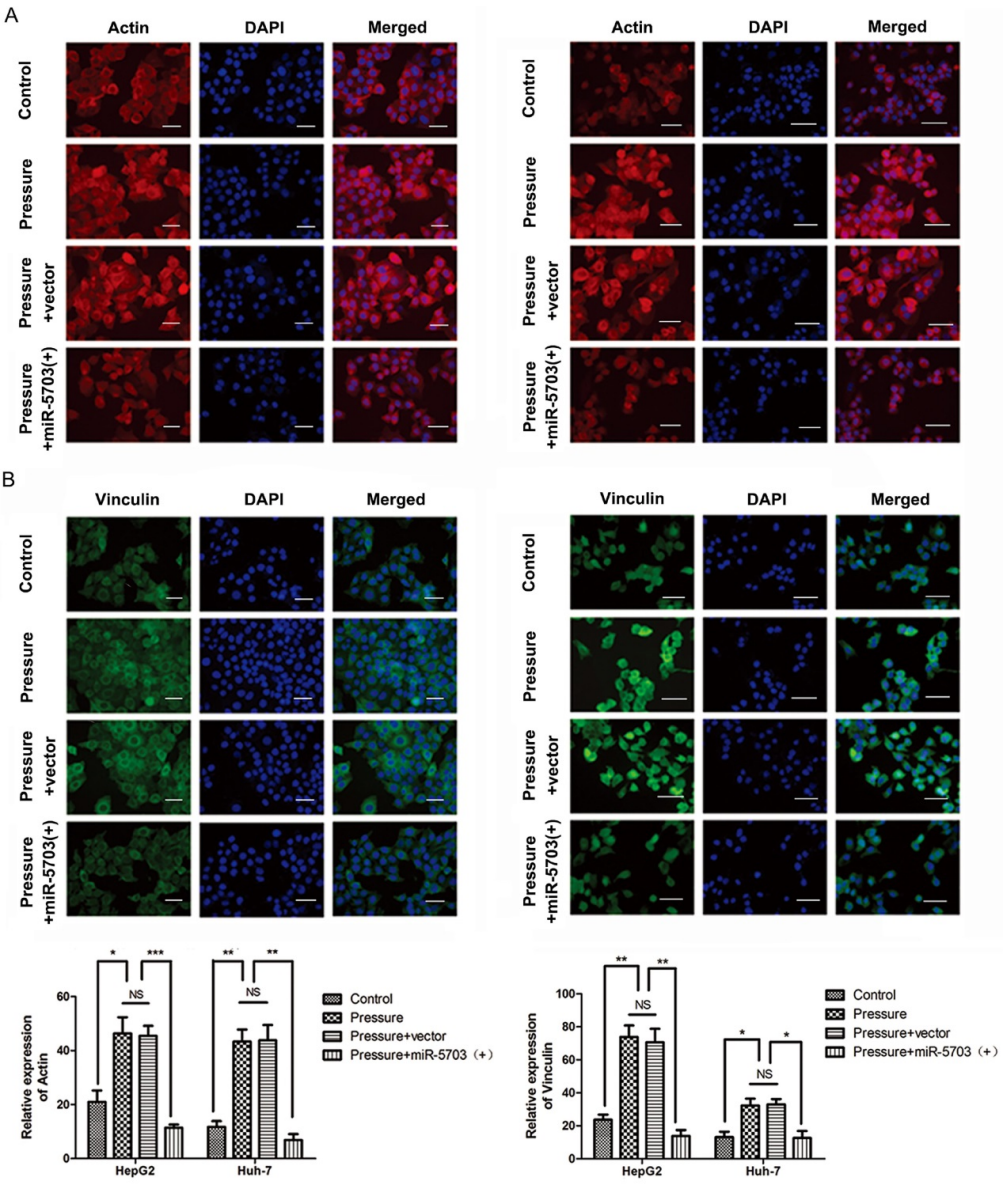
** (A)** Microfilaments of HepG2 and Huh-7 cells transfected with miRNA-5703 mimics were downregulated relative to that of cells transfected with the microRNA negative control (NC) as shown by staining with ghost pencil ring peptide. The quantification of the positive cells is shown on the right. The scale bars are 50 μm. **(B)** The expression of FA protein vinculin was inhibited by miRNA-5703 transfection as measured by immunofluorescence. The scale bars are 50 μm. *NS* indicates the means are not significantly different (P > 0.05), mean ± SD, n = 3. A two-tailed Student's t-test was used. ***P < 0.001, **P < 0.01, *P < 0.05.

The actin microfilaments of HepG2 and Huh-7 cells were stained with the ghost pencil-ring peptide. The results showed that pressure increased the number of cytoskeleton microfilaments, while overexpression of miRNA-5703 significantly reduced it (Fig. **8A**, P < 0.05, P < 0.01, P < 0.001, or P > 0.05). Immunofluorescence showed that the expression of FA protein vinculin was upregulated by pressure, while overexpression of miRNA-5703 significantly inhibited the expression of vinculin in cells under pressure (Fig. **8B,** P < 0.05, P < 0.01, or P > 0.05).

### Overexpression of miRNA-5703 inhibits pressure-induced cell migration and invasion via the SRC-paxillin pathway

**Figure 9:**
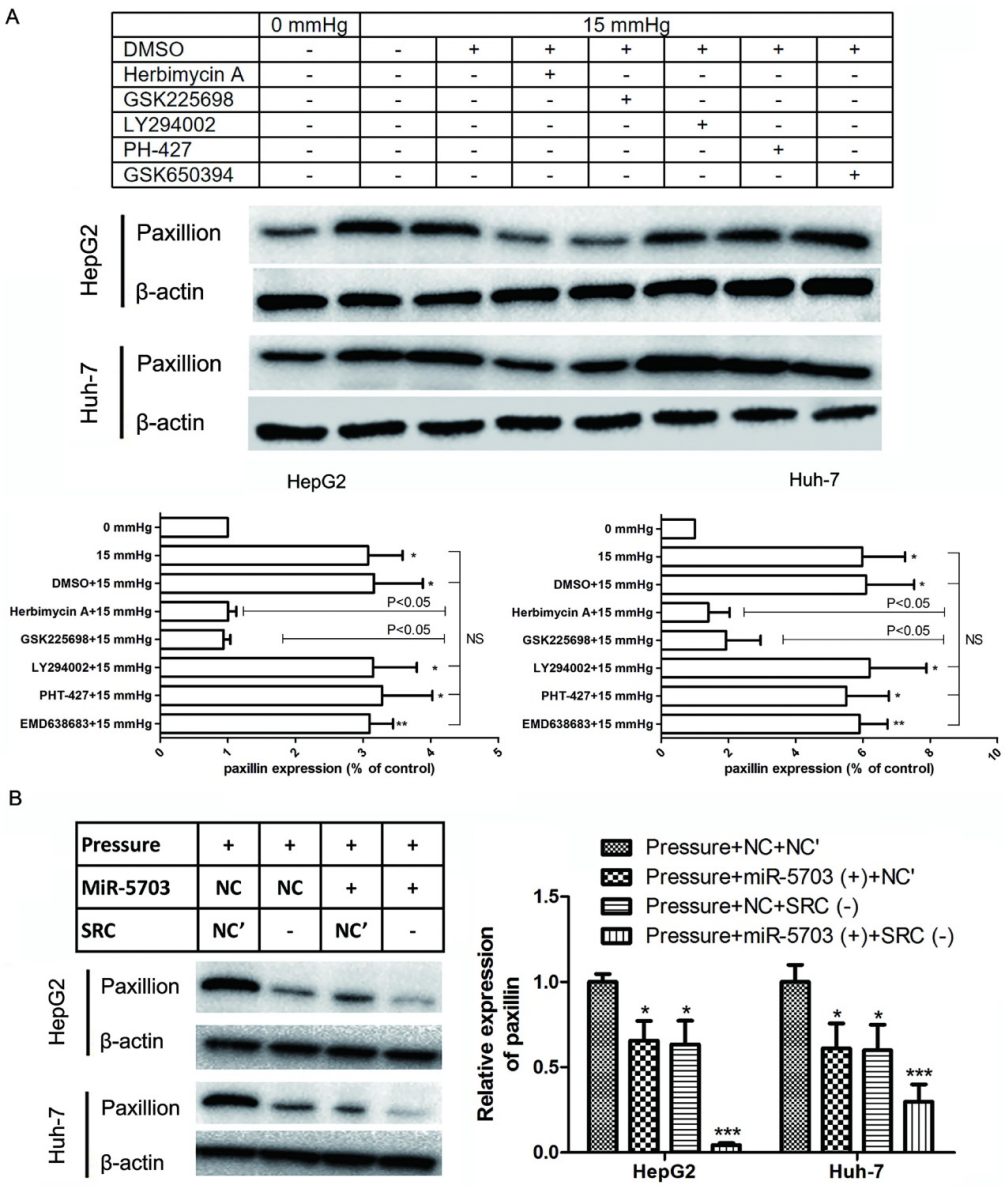
Overexpression of miRNA-5703 inhibited pressure-induced cell proliferation via the SRC-paxillin pathway. **(A)** Overexpression of miRNA-5703 and SRC gene silencing inhibited the expression of paxillin. **(B)** Herbimycin A and GSK2256098 inhibited paxillin expression while LY294002, PHT-427 and EMD638683 did not. β-actin was used as a loading control. Mean ± SD, n = 3. A two-tailed Student's t-test was used. ***P < 0.001, *P < 0.05.

Under 15 mmHg of pressure, herbimycin A and GSK2256098 inhibited paxillin expression (P < 0.05), while LY294002, PHT-427, and EMD638683 had no significant effect on paxillin expression. This indicated that 15 mmHg pressure loading promoted the expression of SRC and FAK in HepG2 and Huh-7 cells, and it remarkably upregulated the expression of paxillin (Fig. **9A**, P < 0.05, or P < 0.01). Overexpression of miRNA-5703 or knockdown of SRC inhibited the expression of paxillin, which suggests that this may be the mechanism by which miRNA-5703 in inhibits tumour metastasis (Fig. **9B**, P < 0.05, or P < 0.01).

**Figure 10:**
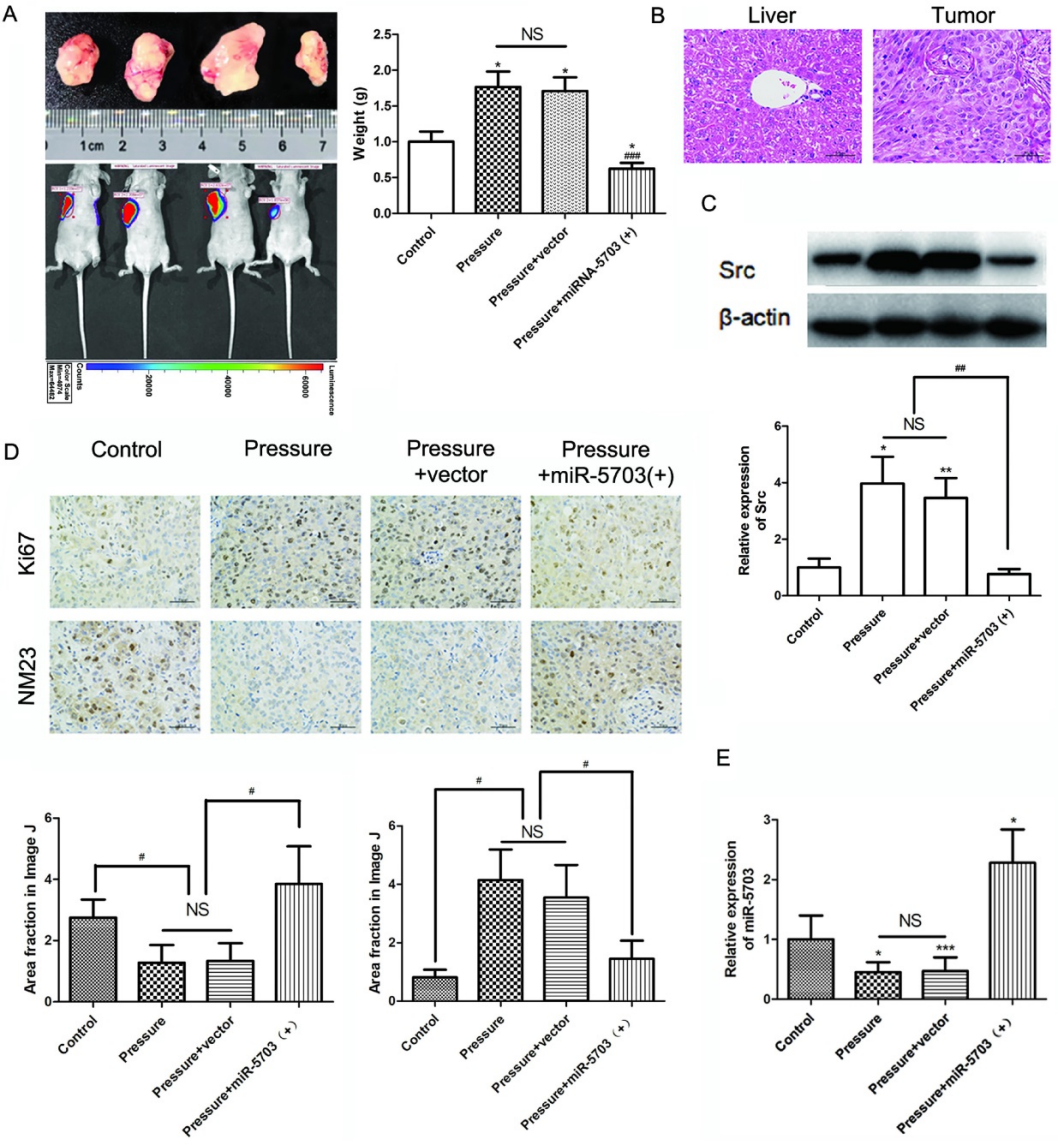
The effect of pressure on the growth and metastasis of liver cancer was verified in nude mice. **(A)** Overexpression of miRNA-5703 inhibited the pressure-induced tumour growth in subcutaneous MHCC97H implanted nude mice. The nude mice were dissected and the subcutaneous tumours were taken out. The weight of the tumours under the four conditions were measured. **(B)** HE staining of normal liver tissues and tumour tissues from subcutaneous MHCC97H implanted nude mice. The scale bars are 50 μm.** (C)** The expression of SRC in tissues from nude mice was detected by western blotting, and β-actin was used as a loading control. **(D)** Immunohistochemical assays showed that overexpression of miRNA-5703 inhibited the expression of Ki67 and upregulated the suppression activity of NM23 in subcutaneous tumours in nude mice. Representative photographs were taken at ×400 magnification. The scale bars are 25 μm. **(E)** The expression of miRNA-5703 in tumours under the four different conditions was detected by RT-qPCR. *NS* indicates the means are not significantly different (P > 0.05), mean ± SD, n = 3. A two-tailed Student's t-test was used. Vs Control: ***P < 0.001, **P < 0.01, *P < 0.05; vs Pressure: ^###^P < 0.01, ^##^P < 0.01, ^#^P < 0.05.

The MHCC97H cells were divided into four groups based on experimental conditions: control (conventional culture), pressure (15 mmHg, 24 h), pressure + vector (15 mmHg, 24 h + vector), and pressure + miRNA-5703 (+) (15 mmHg, 24 h + miRNA-5703 over-expression). The cells were injected subcutaneously into mice and imaged *in vivo* 10 d post-injection. We found that tumour size and weight increased in the pressure loaded cell group, but decreased in the miRNA-5703 overexpression group (Fig. **10A**, P < 0.05, P < 0.001, or P > 0.05). Hematoxylin-eosin (HE) staining of liver cancer and normal liver tissue is shown in Fig. **10B**. The results of the western blot suggested that pressure-upregulated SRC was suppressed in the miRNA-5703 overexpression group (Fig. **10C**, P < 0.05, P < 0.01, or P > 0.05).

**Figure 11:**
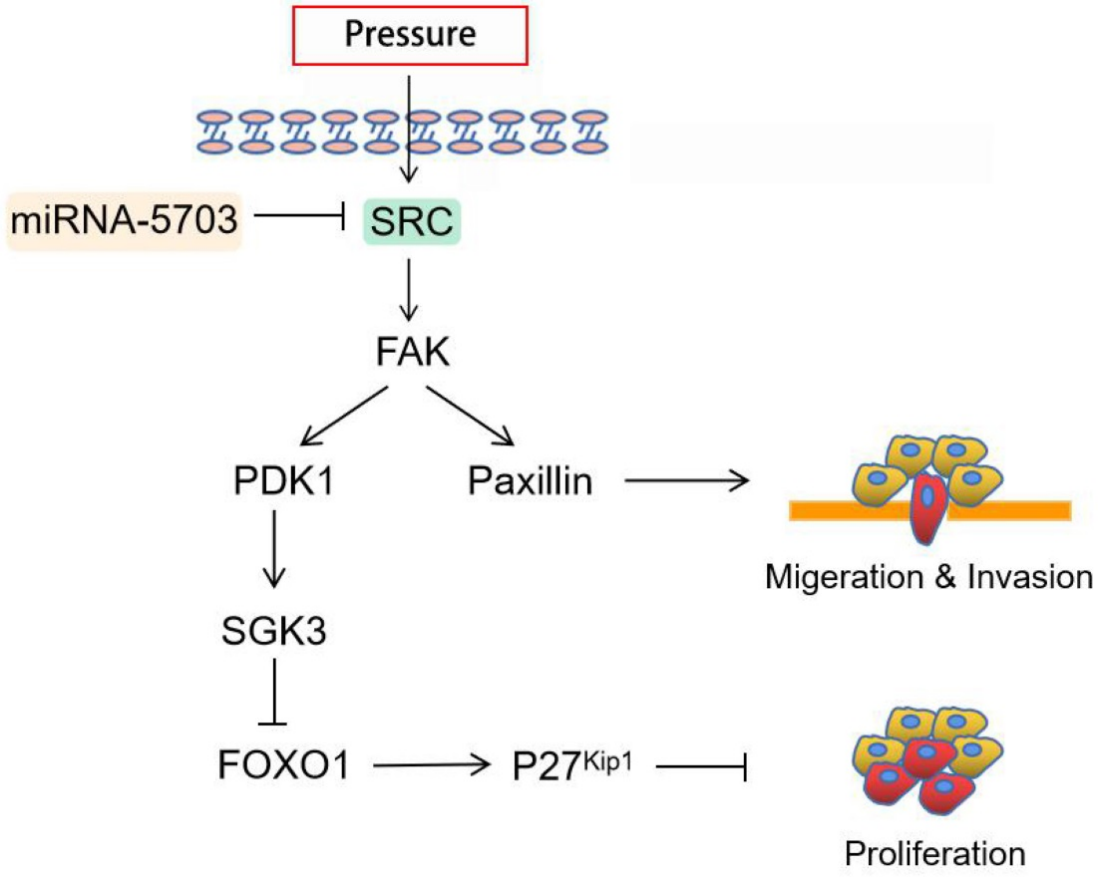
Schematic diagram summarizing how pressure-responsive miRNA-5703 promotes the growth and metastasis of liver cancer by inhibiting SRC expression.

The immunohistochemical assay showed that the expression of Ki67 was upregulated in tumours in the pressurised group and downregulated significantly in tumours in the miRNA-5703 overexpression group, and the expression of NM23 had the opposite pattern (Fig. **10D**, P < 0.05, or P > 0.05). Ki67 is an indispensable antigen in cell proliferation and is associated with mitosis. In clinical practice, Ki67 is used to label cells in the proliferation cycle 33. NM23 is highly expressed in well-differentiated tumours, and it inhibits tumour cell metastasis. It is also negatively correlated with lymph node metastasis. In the clinic, the detection of NM23 gene expression by immunohistochemistry is an important method for judging the metastatic ability of tumours 34. The expression of miRNA-5703 in tumours of the four groups was assessed by RT-qPCR (Fig. **10E**, P < 0.05, P < 0.001, or P > 0.05). The schematic diagram of cascade reaction is shown in Fig. **11**.

## Discussion

In recent years, mechano-inspired anticancer drugs that target components of transduction and mechanosensitive signalling pathways have emerged. Etaracizumab, Volociximab, and Cilengitide are undergoing clinical trials and are aimed at preventing the pro-metastatic signalling transduction associated with integrin-mediated sensing of various mechanical cues in the TME 16. Changes in the liver mechanical microenvironment caused by cirrhosis and PHTN may be involved in the regulation of liver cancer growth and metastasis. However, to date, there has been no research on a drug that targets the mechanical environment of liver cancer which is continuously enveloped by stiff matrix TME and hypertension from the portal system. Our findings suggest that miRNA intervention may prevent tumor promotion from stiffness of the extracellular matrix and suppress the development of liver cancer. These findings provide novel information that can potentially be utilized for the development of new drugs.

SRC was found to be the conjunct upstream protein of the top 3 enriched pathways (FA pathway, FOXO pathway, and PI3K pathway) using GO analysis of the 1,309 predicted target genes of miRNA-5703. Consistent with previous observations in human colon cancer cell lines and breast cancer cell lines 35-38, reducing SRC or inhibiting the FAK/PI3K axis blocked pressure-stimulated cell adhesion in liver cancer cell lines. The deformation of the liver cancer cell membrane caused a pressure-activated expression of SRC and pSRC (Y418), which could bind FA leading to the up-regulation of FAK and pFAK (Y397) and boosting the function of FA 39. Therefore, pressure not only upregulated the expression of total SRC and FAK, but also promoted their phosphorylation, which maximised their activation effect.

Pressure-responsive miRNA-5703, which was identified by miRNA microarray, was predicted to target SRC. The expression of miRNA-5703 in liver cancer tissues of patients with PHTN was significantly lower than that in patients without PHTN, while the expression of SRC had the opposite pattern. *I*ncreased translation of SRC *in vivo* activates downstream FAK and PI3K, which inactivate FOXOs by activating PDK1 to phosphorylate SGK3, suppressing FOXO1 which could inhibit P27^kip1^ and cyclin D1, and ultimately promotes cell proliferation. SRC activates cell motility associated proteins (vinculin, paxillin, and actin), which are important for the normal function of the cytoskeleton. The C-terminal region of paxillin contains four lim domain (LIM) domains, which anchor paxillin to FA 40. The N-terminal region of paxillin is rich in protein-protein interaction sites, leading to its binding to a variety of proteins, including tyrosine kinases such as SRC and FAK 41, structural proteins such as vinculin and actopaxin, and actin regulators 42. Our study found that physical distortion of the cytoskeleton transfers mechanical loads through actin- and microtubule-associated molecules to initiate intracellular signaling, and overexpression of miRNA-5703 may counteract this effect.

Our pressure-induced subcutaneous tumour-forming model of mice is different from that of liver cancer in the context of PHTN in cirrhosis. However, previous studies have suggested that it is feasible to implant cancer cells or stem cells loaded with pressure 43 or shear stress 44, 45 *in vitro* into nude mice and observe tumour formation *in vivo* and detect the expression of related proteins. Based on previous studies, we used 3D cell culture technology to mix cells into biogels to achieve a better simulation of the tumour mechanical microenvironment *in vivo*. Tumour size increased normally after implantation, and the protein expression in each group was significantly different.

Our study has some limitations. Although the role of pressure in promoting the migration and invasion of hepatoma cells has been fully confirmed *in vitro*, we only detected the expression of NM23 in subcutaneous tumours *in vivo*. *In situ* tumourigenesis in nude mice is necessary to further explore the effect of pressure on liver cancer metastasis, and further studies are needed. In addition, whether the cancer-promoting effect of pressure is related to membrane channel proteins is still unknown. Our previous microarray results confirmed that the mRNA levels of integrin subunits (αv, α3, α6, α11, β4, β6, and β7) was upregulated in pressurised hepatoma cells (GEO database: GSE120194) 14. Integrins have been studied extensively in the mechanical signalling of cancer cells and cancer-associated fibroblasts, but not in liver cancer. In addition, the iron channel protein PIEZO2 and its target protein NOTCH1/2 were upregulated (GEO database: GSE120194) 14 PIEZO is a mechanically sensitive channel protein that has recently been implicated in the development and progression of various cancers 46. The function of these membrane proteins and whether pressure-responsive miRNAs act on them requires further study.

## Supplementary Material

Supplementary figures.

## Figures and Tables

**Table 1:** Primer sequences of miRNA-5703 used for reverse transcription‑quantitative polymerase chain reaction.

Gene name	Forward primer sequence
hsa-miR-5703	5'-AGGAGAAGTCGGGAAGGT-3'

**Table 2:** Primer sequences of mRNAs used for reverse transcription‑quantitative PCR.

Gene name	Forward primer	Reverse primer
PTK2	5'-GCTTACCTTGACCCCAACTTG-3'	5'-ACGTTCCATACCAGTACCCAG-3'
PIK3R1	5'-AGCATTGGGACCTCACATTACACA-3'	5'-ACTGGAAACACAGTCCATGCACATA-3'
SRC	5'-GACAGGCTACATCCCCAGC-3'	5'-CGTCTGGTGATCTTGCCAAAA-3'
SGK3	5'-ACAGTCCAAAACACCAGTCAG-3	5'-TCTGTGAGGTAGAGTGTAGCTTC-3'
FOXO1	5'-TGATAACTGGAGTACATTTCGCC-3'	5'-CGGTCATAATGGGTGAGAGTCT-3'
CDKN1B	5'-ATCACAAACCCCTAGAGGGCA -3'	5'-GGGTCTGTAGTAGAACTCGGG -3'
PDPK1	5'-TTCCGAGCTGGAAACGAGTAT-3'	5'-GGTCTCTTGCCTTAGGGAAGAA-3'

## Abbreviations

BCLC: Barcelona Clinic Liver Cancer
BSA: bovine serum albumin
CCK-8: Cell Counting Kit-8
CDKN1B, P27^Kip1^: Cyclin dependent kinase inhibitor 1B
2D: 2-dimensional
3D: 3-dimensional
FOXO1: Forkhead box O1
FAK: Focal adhesion kinase
FA: Focal Adhesion
GAPDH: Glyceraldehyde 3-phosphate dehydrogenase
HE: Hematoxylin-eosin
LIM: Lim domain
PBS: phosphate-buffered saline
PI3K, PIK3R1: Phosphatidylinositol 3-kinase
PDPK1, PDK1: Phosphoinositide dependent protein kinase 1
PHTN: Portal hypertension
PTK2: Protein tyrosine kinase-2
RT-qPCR: Quantitative reverse transcription polymerase chain reaction
SD: Standard deviation
SGK3: Serum/glucocorticoid regulated kinase-3
SRC: Sarcoma gene
TME: Tumour microenvironment
