# Supplementary material for: Overexpression of pressure-responsive miRNA-5703 inhibits pressure-induced growth and metastasis of liver cancer
Source: J Cancer. 2022 Jan 1;13(1):325–42. doi: 10.7150/jca.64926 (PMC8692678; doi:10.7150/jca.64926)
Supplement: Supplementary file 1 — Supplementary figures. [file jcav13p0325s1.pdf]

Fig. S1

Western blotting was used to detect the expression of the SRC-FAK-FOXO1 pathway proteins in HepG2 cells, which was shown in Fig. 4A, and the histograms were drawn based on the grey analysis of the bands. *NS* indicates the means are not significantly different ($P > 0.05$), mean ± SD, $n = 3$. A two-tailed Student's t-test was used. ***$P < 0.001$, **$P < 0.01$, *$P < 0.05$.

Fig. S2

Western blotting was used to detect the expressions of the SRC-FAK-FOXO1 pathway proteins in Huh-7 cells, which were shown in Fig. 4B, and the histograms were drawn based on the grey analysis of the bands. *NS* indicates the means are not significantly different ($P > 0.05$), mean ± SD, $n = 3$. A. two-tailed Student's t-test was used. ***$P < 0.001$, **$P < 0.01$, *$P < 0.05$.

Fig. S1

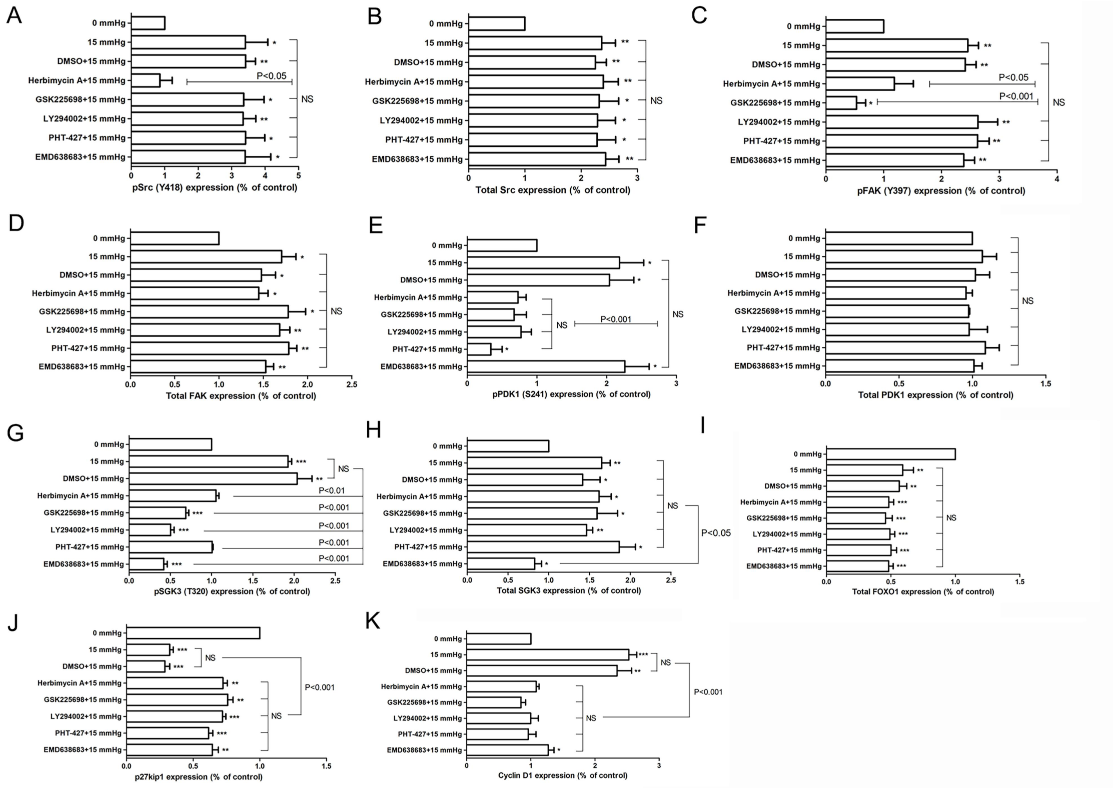
A
0 mmHg
15 mmHg
DMSO+15 mmHg
Herbimycin A+15 mmHg
GSK225698+15 mmHg
LY294002+15 mmHg
PHT-427+15 mmHg
EMD638683+15 mmHg
P<0.05
NS
pSrc (Y418) expression (% of control)
B
Total Src expression (% of control)
C
P<0.05
P<0.001
pFAK (Y397) expression (% of control)
D
Total FAK expression (% of control)
E
P<0.001
pPDK1 (S241) expression (% of control)
F
Total PDK1 expression (% of control)
G
P<0.01
P<0.001
pSGK3 (T320) expression (% of control)
H
P<0.05
Total SGK3 expression (% of control)
I
Total FOXO1 expression (% of control)
J
P<0.001
p27kip1 expression (% of control)
K
P<0.001
Cyclin D1 expression (% of control)

Fig. S2

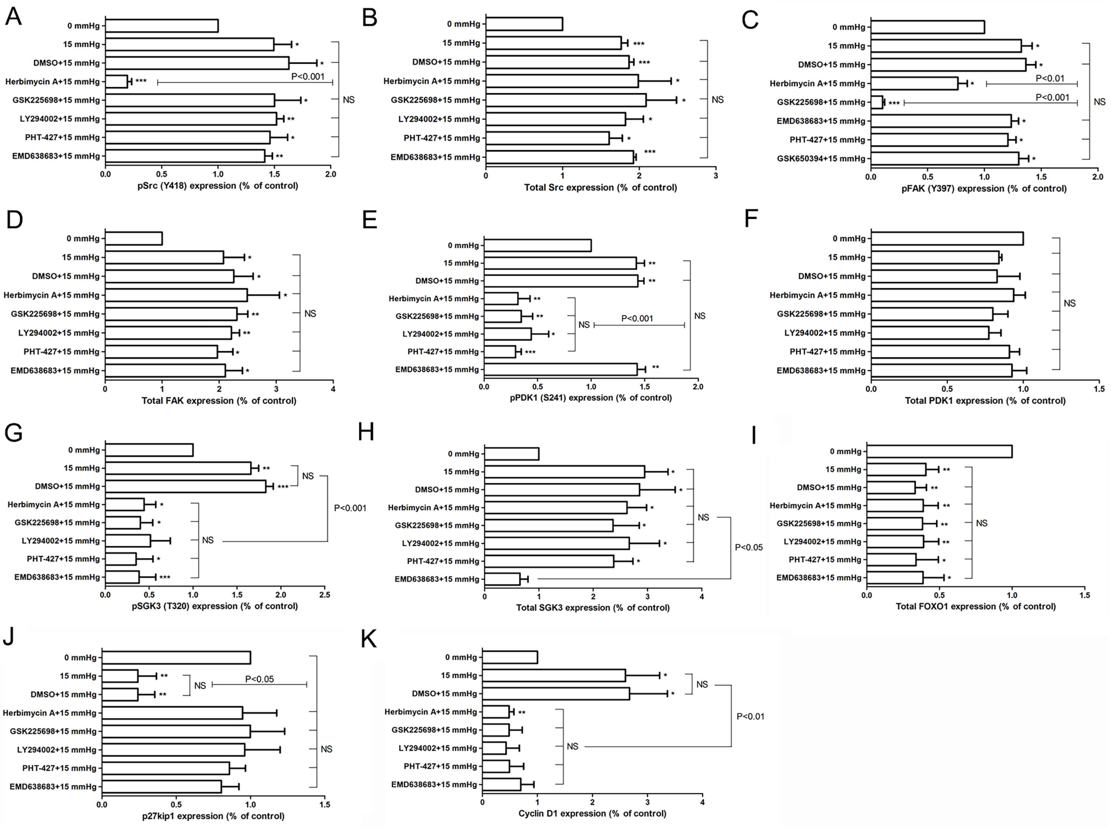
A
0 mmHg
15 mmHg
DMSO+15 mmHg
Herbimycin A+15 mmHg
GSK225698+15 mmHg
LY294002+15 mmHg
PHT-427+15 mmHg
EMD638683+15 mmHg
P<0.001
NS
pSrc (Y418) expression (% of control)
B
Total Src expression (% of control)
C
GSK650394+15 mmHg
P<0.01
pFAK (Y397) expression (% of control)
D
Total FAK expression (% of control)
E
pPDK1 (S241) expression (% of control)
F
Total PDK1 expression (% of control)
G
pSGK3 (T320) expression (% of control)
H
P<0.05
Total SGK3 expression (% of control)
I
Total FOXO1 expression (% of control)
J
p27kip1 expression (% of control)
K
Cyclin D1 expression (% of control)
